# Comparison between Two Assessment Tests for Oral Hygiene: Adenosine Triphosphate + Adenosine Monophosphate Swab Test and Bacteria Number Counting by Dielectrophoretic Impedance Measurement

**DOI:** 10.3390/dj7010010

**Published:** 2019-02-01

**Authors:** Yuki Iwawaki, Yuki Muraoka, Hiroaki Higashiyama, Takahiro Kishimoto, Lipei Liu, Takaharu Goto, Tetsuo Ichikawa

**Affiliations:** Department of Prosthodontics and Oral Rehabilitation, Tokushima University, Graduate School of Biomedical Sciences, 3-18-15, Kuramoto, Tokushima 770-8504, Japan; iwawaki.yuuki.1@tokushima-u.ac.jp (Y.I.); muraoka.yuki@tokushima-u.ac.jp (Y.M.); chei2010@amail.plala.or.jp (H.H.); c301751010@tokushima-u.ac.jp (T.K.); c301751017@tokushima-u.ac.jp (L.L.); ichi@tokushima-u.ac.jp (T.I.)

**Keywords:** oral bacteria, ATP + AMP swab test, luciferase assay, bacteria counting, dielectrophoretic impedance measurement

## Abstract

Objective assessments of oral hygiene are important to prevent oral and systemic diseases. Two objective assessment tests are available to assess oral hygiene; (1) the adenosine triphosphate (ATP) + adenosine monophosphate (AMP) swab test, which incorporates a luciferase assay and (2) a bacteria count using the dielectrophoretic impedance measurement (DEPIM) method. In this study, we compared the two tests using a subjective visual assessment by professional clinicians and investigated the clinical significance of these tests. Twenty-seven young participants (mean age 26.3 ± 3.2 years) and twenty-seven older participants (mean age 75.1 ± 5.9 years) were recruited. Oral bacteria were sampled from three areas, including the tongue dorsum, the buccal mucosa, and the faucal mucosa, and saliva was obtained using a cotton swab. The amount of ATP + AMP and the number of bacteria were measured by each specific apparatus. Additionally, one examiner assessed the overall condition of oral hygiene using the visual analog scale (VAS). In the ATP + AMP swab test, the means were highest in saliva. For the bacteria count, the means were higher in the tongue dorsum and saliva and lower in the faucal and buccal mucosa. The results of the subjective assessment of oral hygiene indicated that the VAS-value was 3.78 ± 0.97 for the young group and 3.35 ± 0.81 for the older group. No significant difference was observed between the two groups. Additionally, no significant relationship between the values of the ATP + AMP swab test and the bacteria count was found for any of the four sample sites. In the older group, the subjective assessment of oral hygiene was significantly correlated with the values of the ATP + AMP swab test (multiple correlation coefficient = 0.723, *p* = 0.002). In conclusion, the values provided by the ATP + AMP swab test were not always correlated to the bacteria count. The results of this study suggest that the subjective assessment of oral hygiene was more highly correlated with the results of the ATP + AMP swab test, as compared to the bacterial count assay.

## 1. Introduction

Poor oral hygiene, a condition associated with a significantly increased number of oral microorganisms within the oral cavity, has been discussed as a risk factor that may contribute to dental caries and periodontal disease development [1,2]. Aspiration pneumonia is a major cause of morbidity and mortality in the elderly. Multiple risk factors for aspiration pneumonia have been identified, such as aging, cerebrovascular disorders, and dementia. Poor oral hygiene has also been suggested as a direct cause of this disease [3,4]. Additionally, it has been reported that poor oral hygiene can lead to a high incidence of postoperative pneumonia and various infections in addition to aspiration pneumonia [5,6,7].

During a dental check-up, plaque accumulation on teeth is assessed and gingiva and tongue assessments of oral hygiene, such as the papillary marginal attached index, gingival index, plaque control record, and tongue plaque index are also performed [8,9,10]. These assessments are traditionally used in dental practice. However, they may not provide precise and objective numerical assessments and they also fail to include assessments of saliva and oral mucosa. To evaluate cleaning instructions or interventional outcomes in the context of oral hygiene, objective assessments are required. Additionally, effective assessments require a certain degree of skill. The cultivation of oral bacteria is available for objective assessments, but the smear culture is time-consuming and labor-intensive. The spiral plater and selective medium technique, however, could greatly accelerate the quantification of viable bacteria. Recently, innovative objective tests that are also easily performed have been proposed to assess oral hygiene. One method is dielectrophoretic impedance measurement (DEPIM), which is used to count the number of bacteria automatically [11,12,13], and the other is the adenosine triphosphate (ATP) + adenosine monophosphate (AMP) swab test [14]. Both tests allow for rapid and immediate assessments, as opposed to relying on bacterial cultivation methods and various visual indices. In many past studies, a significant correlation has already been reported between viable count data and data produced by the DEPIM method for several indices of oral hygiene, including tongue dorsum or buccal mucosa [12,15,16]. Although a significant correlation between ATP + AMP values and viable count data in saliva has also been reported in past studies [14,17], the relationship between the ATP + AMP values of the oral cavity and saliva or aging has not yet been comprehensively studied. In addition, it is unknown if the relationship between the values generated by the two tests is clinically useful. Further information is required to effectively use the tests as objective assessments of oral hygiene for dental treatment and care.

The purpose of this study was to clarify the clinical significance of the ATP + AMP swab and the DEPIM test for counting bacteria number. This was achieved by examining the oral hygiene of young and older people using these two tests and comparing them with a subjective visual assessment of oral hygiene status.

## 2. Materials and Methods

### 2.1. Participants

Twenty-seven young participants (mean age = 26.3 ± 3.2 years, 16 males and 11 females) and twenty-seven older participants (mean age = 75.1 ± 5.9 years, 12 males and 15 females) were recruited from the Faculty of Dentistry and from a group of older patients who were undergoing regular maintenance at the prosthodontics division of Tokushima University Hospital, respectively. Regarding the dentition status, the young group subjects exhibited normal natural dentitions, and the dental status of the elderly group subjects was typically restored with restorations and/or dentures. The present study was approved by the Ethics Committee of the Tokushima University Hospital (approval number: 2338), and all participants signed a written informed consent form prior to study participation.

### 2.2. ATP + AMP Swab Test

**Figure 1:**
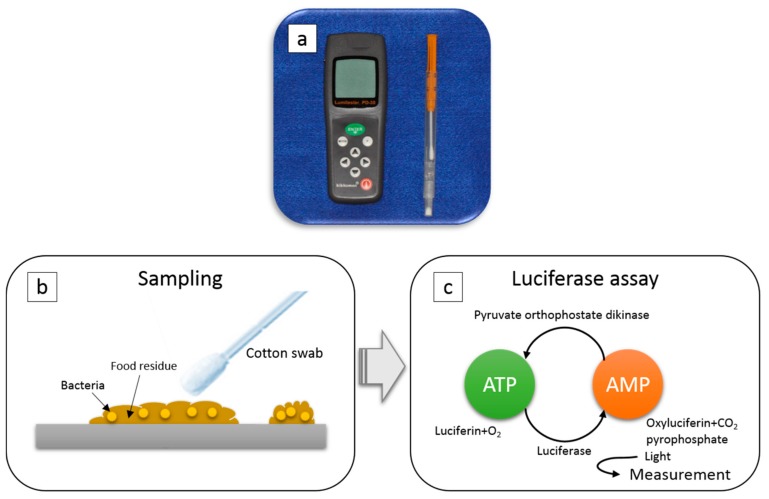
ATP + AMP swab test: (**a**) The portable analysis device Lumitester, (**b**) The test area was wiped using a special cotton swab, (**c**) The degree of ATP + AMP contamination was detected by wiping the test object and based on the “firefly principle”, in which ATP reacts with luciferin in the presence of luciferase to generate AMP and emits light. Using this ATP + AMP swab test, relative light emission (RLU) can be measured.

Figure 1 describes the ATP + AMP swab test. ATP is a substance that acts as an energy source for all living organisms and AMP is derived from ATP through processing. This test detected the degree of ATP + AMP contamination by wiping the test object and measuring using the “firefly principle”, a process in which ATP reacts with luciferin in the presence of luciferase to generate AMP and emit light [18]. In the ATP + AMP swab test, relative light emission (RLU) can be measured. Additionally, luminescence is stabilized and the amount of AMP present at the sampling site can be measured when AMP is converted again to ATP by pyruvate orthophosphate dikinase (PPDK). The resulting intensity depends on the amount of biological materials present, such as viable and killed bacteria and food residue. The ATP + AMP swab test was performed using a Lumitester (PD-30, Kikkoman, Japan), which provides rapid hygiene monitoring applications at a low cost and with ease of operation. The ATP + AMP value was automatically calculated after inserting the swab into the Lumitester. A special cotton swab was used for each sampling site.

### 2.3. Counting Bacterial Number Using DEPIM

**Figure 2:**
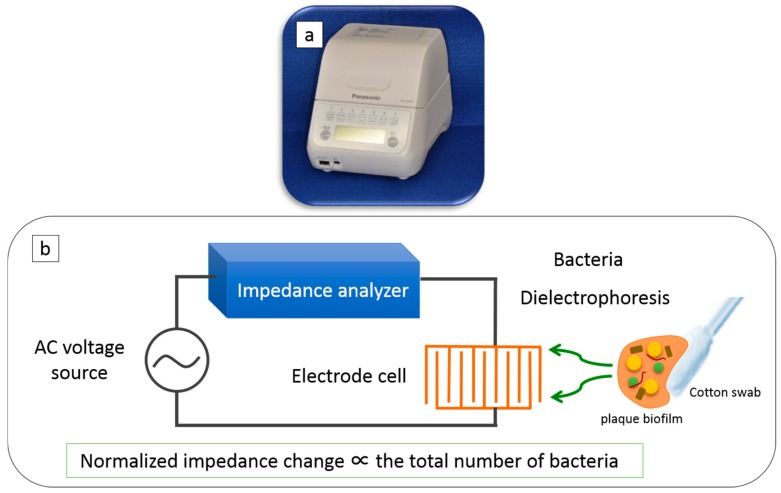
Dielectrophoretic impedance measurement (DEPIM) method: (**a**) Bacterial counter based on the DEPIM method, (**b**) Bacteria were captured by dielectrophoresis (DEP) through the integration of a microelectrode chip, an AC voltage source to induce DEP force, and a portable impedance measurement instrument).

The apparatus to count the bacteria number was based on the DEPIM method, as seen in Figure 2. The bacteria were captured by dielectrophoresis (DEP) through the integration of a microelectrode chip, an AC voltage source to induce DEP force, and a portable impedance measurement instrument that enabled rapid and automated chair-side oral bacterial inspection. The test area was wiped using a special cotton swab in a manner identical to that of the ATP + AMP swab test. Bacterial numbers were automatically counted by inserting the swab into the device. A special cotton swab was used for each sampling site.

### 2.4. Sampling of Oral Bacteria

**Figure 3:**
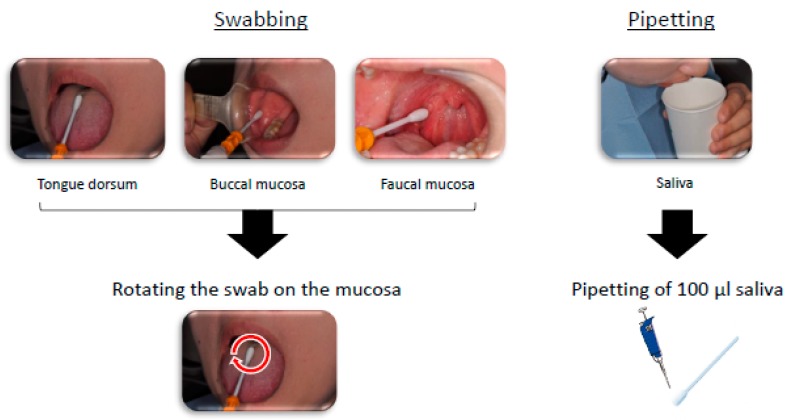
Sampling of oral bacteria in three regions and saliva.

All assessments were performed chair-side in the hospital without any instruction prior to the appointment. The sampling was performed after each participant was instructed to rinse their mouth quickly with water. Oral bacteria were swabbed from the tongue dorsum, buccal mucosa, and faucal mucosa by rotating the sterilized specific cotton swab on each region. Participants were asked to spit in a paper cup to collect saliva. Subsequently, 100 μL of the saliva was transferred to a cotton swab by pipetting, as indicated in Figure 3. The cotton swab was inserted into each measuring device according to the operation manual and measured values were obtained.

### 2.5. Subjective Assessment of Oral Hygiene

One examiner with eight years of dental experience observed the oral cavity of each participant and subjectively assessed the overall condition of oral hygiene according to the visual analog scale (VAS), which is a straight horizontal line. The ends were defined as the extreme limits of oral hygiene conditions, especially in the tongue, buccal mucosa, and gingiva, and measurements were oriented from the left (1) to the right (5). In this study, a modified VAS was used by referencing Löe’s index [19]. The classification included Score 1 (abundance of plaque on every surface of teeth, tongue, buccal mucosa, and gingiva), Score 2 (abundance of plaque on any one or more than one surface of teeth, tongue, buccal mucosa, and gingiva, but not on every surface), Score 3 (moderate plaque accumulation on any one or more than one surface of teeth, tongue, buccal mucosa, and gingiva, but not on every surface), Score 4 (plaque only visible after using the probe on any one or more than one surface of teeth, tongue, buccal mucosa, and gingiva, but not on every surface), and Score 5 (no plaque in teeth, tongue, buccal mucosa, and gingiva). The examiner was asked to place a line perpendicular to the VAS line after understanding the meaning of the rating indexes.

### 2.6. Statistical Analysis

The Mann–Whitney U test was used to compare the values from the ATP + AMP and bacterial count tests of the young and older group and multiple regression analysis was used for statistical analysis. For multiple regression analysis, the subjective assessment of oral hygiene was used as the response variable and each value of the ATP + AMP swab test and the bacterial count measurement was used as the explanatory variable in all sampling sites selected. All statistical analyses were conducted using a significance level of 0.05 as determined by the SPSS^®^ software (version 24.0, IBM Corp., Armonk, NY, USA).

## 3. Results

The means and standard deviations of the remaining teeth numbers were 28.8 ± 1.6 in the young group and 12.6 ± 6.7 in the older group, respectively. In the older group, 24 subjects (89%) wore removal dentures.

**Figure 4:**
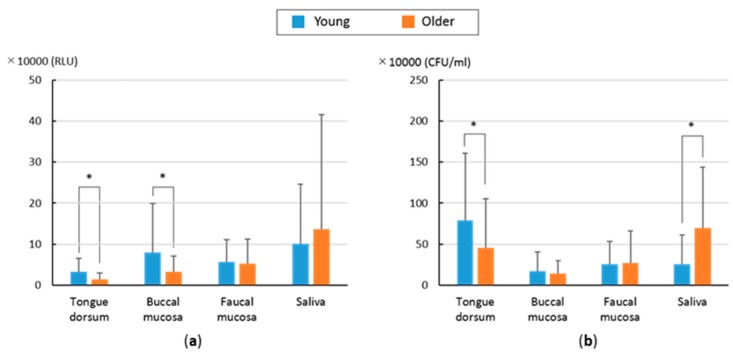
Means and standard deviations of test values in four sampling areas. (* *p* < 0.05, Mann-Whitney U test.). (**a**) ATP + AMP swab test; (**b**) Bacteria number counting using DEPIM.

Figure 4 provides the means and standard deviations of the test values obtained from each sampling area. For the ATP + AMP swab test, the means were highest in the saliva. For the bacterial count assays, the means were higher in the tongue dorsum and saliva and lower in the faucal and buccal mucosa. The magnitude relation profiles of four sampling sites used in each test were dissimilar between the young and older groups and the profiles were different between the two tests. For the ATP + AMP swab test, the means of the data derived from the young group were significantly higher than those of older group in the tongue dorsum and the buccal mucosa. For the bacterial count, the mean of the young group in the tongue dorsum was significantly higher than that of the older group. Conversely, the mean bacterial count in saliva was significantly higher in the older group in comparison to the young group. 

For the subjective assessment of oral hygiene, the mean and standard deviation of the VAS-values were 3.78 ± 0.97, with a range of 2 and 5 (mode = 3) in the young group and 3.35 ± 0.81 with the range of 1.5 and 4.5 (mode = 4) in the older group. No significant difference was found between the two groups.

**Figure 5:**
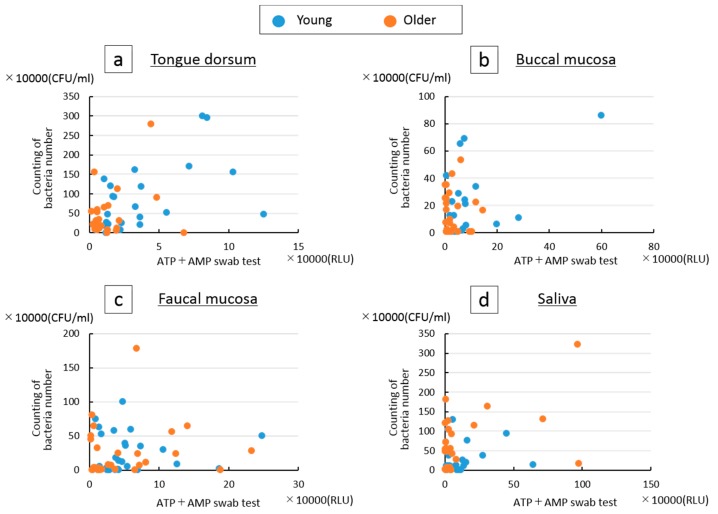
Scatter charts of two test values in four sampling areas: (**a**) tongue dorsum, (**b**) buccal mucosa, (**c**) faucal mucosa, (**d**) saliva.

Figure 5 shows the scatter charts of two test values derived from the four sampling areas. No significant relationship between the values was found among the four sample areas.

Using multiple regression analysis, we examined the assessment of four areas in each test relative to the subjective visual assessment by professional clinicians of oral hygiene. In the older group, the subjective assessment of oral hygiene was significantly (multiple correlation coefficient = 0.723, *p* = 0.002) predicted by the values obtained from the ATP + AMP swab test at all sample sites. No significant relationship was found between the subjective assessment and bacterial count assay results in either the young and older groups.

## 4. Discussion

The standard culture-based plate counting method has conventionally been used to quantify microflora; however, this process is time-consuming and labor-intensive. A rapid oral bacteria detection apparatus using the DEPIM method has been developed to detect total oral bacteria numbers on the tongue dorsum within one minute at chair-side. It is known that the swab pressure during sampling influences the detected bacterial numbers, and as a result, a special jig to maintain a regular swab pressure must be attached to the system. Kikutani et al. reported that an oral bacteria count of 10^8.5^ colony-forming units/mL saliva was identified as a risk factor for pneumonia onset in an elderly person requiring care [20]. Bacterial counting using the DEPIM method has been validated through clinical research and there are significant associations between the bacterial count value and clinical symptoms [21,22]. However, the relationship between the DEPIM value and standard plate counting varies depending on the bacterial species [12] and the DEPIM cannot identify bacterial species. This point is a limitation of the method.

The ATP + AMP swab test has been used to determine the cleanliness of cooking areas and public spaces [23]. It can easily quantify biological materials, including bacteria and food residue, and is effective for rapid assessments in clinical situations that require a bacteria count. There is, however, insufficient information concerning the association of the ATP + AMP swab test with clinical symptoms. Additionally, the cut-off values for good/poor hygiene have not been directly identified, although the values are relative. In this study, the clinical significance of the test was examined in comparison to the bacterial counting method. The cost should be considered as a negative aspect of both tests, as the measurement device and consumables such as special cotton swabs or measurement chips must be prepared when making a comparison to a visual inspection. In Japan, aspiration pneumonia is a crucial problem among the elderly and such objective assessments for oral hygiene are necessary, as these two tests can be applied to bedridden patients who are unable to visit dental clinics and need home dental care.

In this study, the upper limit of the bacterial counts was approximately 7.9 × 10^5^ CFU/mL. Bacterial counts in saliva were reported to be approximately 10^8–9^/mL [24]. However, bacterial counts in the oral cavity have also been reported to be different according to each meal, brushing, sampling method, or testing time. The results of a previous study incorporating the same method as our study were similar to those provided here [25]. Thus, the results of our study have been independently supported.

The magnitude relation profiles of the four sites sampled in each test were not similar between the young and older groups and the profiles were different between the two tests. When direct comparisons were made using the scatter charts, no significant correlation was found, suggesting that these tests did not detect similar oral hygiene conditions. In principle, the two tests are different. The ATP + AMP swab test is influenced by not only the amount of viable and killed bacteria, but also by food residue and epithelial cells. Additionally, a standard value of one was set when the oral bacteria count was less than 10^6^ colony-forming units/mL saliva. Given this, the dynamic range of the ATP + AMP swab test would be larger than that provided by the bacterial count assay, as suggested in the scatter charts.

With regard to the comparison of the young and older groups, the means from the young group were significantly higher than those of the older group in the tongue dorsum and buccal mucosa and this was especially true for the ATP + AMP swab test. One explanation for this is that the subjects in the older group visit dental hospitals for regular dental check-ups and are more oral hygiene-conscious than the young group. In our study, neither the number of times a participant brushed their teeth, nor the time at which individuals brushed their teeth, was standardized. Thus, further study is needed to determine the potential effects of these biases.

The multiple regression analysis suggested that the subjective assessment of oral hygiene was more highly correlated with the results of the ATP + AMP swab test than the bacterial count assays. This may be because the ATP + AMP swab test possesses a wider dynamic range, whilst subjective assessment may also be influenced, not only by plaque adherence, but evidence of food residue and other factors. The results of this study suggest that the ATP + AMP swab test may be applicable for the assessment of oral hygiene, although the association between the ATP + AMP swab test and clinical symptoms has not been as extensively examined as it has been with the bacterial count method.

In conclusion, the values provided by the ATP + AMP swab test are not always correlated to those of the bacterial count assay. The results of this study suggested that the subjective assessment of oral hygiene was more highly correlated with the results of the ATP + AMP swab test, in comparison to the bacterial count assay.
